# Evaluation of health-related quality of life changes in an Australian rapid access chest pain clinic

**DOI:** 10.1186/s12913-024-12135-0

**Published:** 2025-01-02

**Authors:** J Andrew Black, James E. Sharman, Gang Chen, Andrew J. Palmer, Barbara de Graaff, Mark Nelson, Niamh Chapman, Julie A. Campbell

**Affiliations:** 1 Department of Cardiology, Royal Hobart Hospital, 48 Liverpool Street, Hobart, TAS Australia; 2 College of Health and Medicine, Menzies Institute for Medical Research, University of Tasmania, 17 Liverpool Street, Hobart, TAS Australia; 3 Centre for Health Economics, Monash University, 900 Dandenong Rd, Caulfield East, Victoria, Australia; 4 Health Economics Unit, School of Population and Global Health, University of Melbourne, 207 Bouverie Street, Melbourne, VIC Australia; 5 School of Health Sciences, Faculty of Medicine and Health, University of Sydney, Sydney, NSW Australia

**Keywords:** Absolute cardiac risk, Quality of life, Chest pain clinic, Economic evaluation, Cost-utility

## Abstract

**Objective:**

To evaluate the impact of absolute cardiovascular risk counselling on quality-of-life indices within a chest pain clinic.

**Data sources and study setting:**

Primary data was collected at the Royal Hobart Hospital, Australia, between 2014 and 2020.

**Study design:**

Patients attending an Australian chest pain clinic were randomised into a prospective, open-label, blinded-endpoint study over a minimum 12-months follow-up.

**Data collection / extraction methods:**

The SF-36 questionnaire was completed at baseline/follow-up and SF-6D multi-attribute utility instrument’s health state utilities (HSU) were generated using SF-36 responses and the SF-6D’s Australian tariff. SF-6D minimal important difference was 0.04 points. Absolute cardiovascular risk was also stratified into high/intermediate/low-risk categories for exploratory analysis of summary HSUs and dimensional scores. ANZCTR registration number 12617000615381 (registered 28/4/17).

**Principal findings:**

Of *n* = 189 patients enrolled, HSUs were generated for 96% at baseline (intervention *n* = 93, usual care *n* = 88) and 61% at follow-up. There were no statistical differences in age, sex, absolute cardiovascular risk or mean HSU between groups at baseline. Summary HSUs improved more for the intervention group and the median between-group difference exceeded the minimal important difference threshold (intervention 0.16 utility points, control 0.10 utility points). For Intervention patients with high absolute risk (≥ 15%), HSU did not significantly change.

**Conclusions:**

Absolute cardiovascular risk counselling in a chest pain clinic yielded clinically meaningful improvement in health-related quality of life.

**Supplementary Information:**

The online version contains supplementary material available at 10.1186/s12913-024-12135-0.

## Callout box

What is known on this topic: Rapid Access Chest Pain Clinics streamline care and improve clinical outcomes for patients presenting with new onset chest pain. Many do not have active cardiovascular disease, but there is a high prevalence of potentially modifiable risk factors.

What this study adds: Using the SF-6D, we found that absolute cardiac risk counselling in a RACPC improves quality of life.

## Introduction

Cardiovascular disease is the leading cause of death worldwide and imposes a significant health economic burden, including health-related quality of life (HRQoL) burden, on individuals, health payers and society more broadly [1–5]. In Australia in 2017–18, an estimated 1.2 million (6%) adults aged 18 years and over had one or more conditions related to heart or vascular disease based on self-reported data from the Australian Bureau of Statistics (ABS) National Health Survey [6]. The associated direct and indirect health economic costs worldwide are substantial and increasing [3, 7] and patients with concommitant cardiac risk factors generally have poor HRQoL [8]. This has led to an increasing focus on affordable effective preventative strategies to shift the population risk, including the use of prognostic tools and risk scores [9]. Moreover, a health consumer’s understanding of health risks is a key determinant of effective risk communication about health problems; individual’s make decisions about their health under uncertainty, and the influence of risk perception, risk preferences and information processing are crucial in these decision-making processes [10].

Rapid Access Chest Pain Clinics (RACPCs), first utilised in the United Kingdom, provide safe and efficient evaluation of patients with new onset chest pain [11–15]. Patients and referrers report a high degree of satisfaction with this streamlined model of care [16, 17]. Compared to a traditional general cardiology clinic model, RACPC assessment may result in lower rates of emergency department re-attendance [17].

Following assessment in RACPCs, fewer than 15% of patients presenting with chest pain are found to have a cardiac cause for their symptoms [17, 18]. However, patients presenting with chest pain have a high prevalence of risk factors for future cardiovascular events [17]. Generally, the management of underlying risk factors is not a major focus of chest pain clinics and there is potentially a missed opportunity to embed a preventive health role in these clinics. Our group conducted a randomised clinical trial to investigate the effects of absolute risk-guided proactive cardiac risk counselling (intervention group) on subsequent cardiac risk within a RACPC. The details of the trial protocol as well as the clinical outcomes have previously been reported [19]. The primary end point was change in 5-year absolute risk score. Secondary end-points included lipid profile, blood pressure, smoking status, body-mass index (BMI), major adverse cardiac events and global health state utility (HSU) assessed with the SF-6D multi-attribute utility instrument [19]. This study describes in detail the changes in HRQoL amongst trial participants.

Preference-based HRQoL measures such as the SF-6D, EQ-5D, HUI 3 and AQoL-8D are not only patient-reported outcome measures but also relevant for health economic evaluations since they can be used to derive health state utilities (HSUs) [20, 21]. HSUs have also been shown to be independent predictors of patient outcomes, including all-cause mortality and development of complications [22] and clinicians have found that measuring HSU is of benefit to patients regarding clinical assessment, relationships, communication and management [23]. It has been suggested that clinicians could routinely adopt these instruments in their clinical care [24]. For people with complex and chronic disease, capturing and assessing complex physical and psychosocial health needs through patient-reported outcome measures is crucial [25]. Some multi-attribute utility instruments also capture and assess dimensional scores that drive the HSU. These dimensional scores include, for example: ‘social role’, ‘mental health’ and ‘vitality’ (SF-6D); or ‘happiness’, ‘coping’, ‘self-worth’ (AQoL-8D) [20, 25].

To our knowledge, no studies have presented a detailed evaluation of HRQoL using SF-6D HSUs (including stratified for cardiac risk scores) *and* dimensional scores to determine HRQoL impacts for people who receive absolute cardiovascular risk counselling versus usual care in an outpatient cardio-protective cohort without clinically overt cardiac disease. This study used the SF-6D multi-attribute utility instrument to provide a detailed assessment of HRQoL impacts at baseline and follow-up for people who presented with chest pain where cardiac pathology had been excluded yet had a high burden of cardiovascular risk factors (defined as five-year absolute risk ≥ 8%). Patients were: 1. counselled on their absolute cardiac risk (intervention), prior to discussing a proactive strategy aimed at reducing this risk; or 2. counselled on individual cardiac risk factors at the discretion of the treating clinician (best practice usual care). All participants were invited to complete the SF-36 questionnaire at baseline and follow-up and therefore the SF-6D tool was used in the evaluation of HRQoL outcomes.

## Methods

### Prespecified quality of life analysis within a clinical trial

This study is a prespecified analysis of SF-6D HSUs and dimensional scores within a prospective, randomized, open-label, blinded-endpoint study to evaluate the benefit of an absolute-risk guided proactive risk factor management strategy over best practice usual care in an Australian tertiary hospital RACPC. The trial was registered (ACTRN12617000615381) and approved by the University of Tasmania’s Human Research Ethics Committee (H0014029). The Rapid Access Chest Pain Clinic research programme has been approved by the Tasmanian Health Department’s Research Governance Unit.

#### Patient recruitment

The study protocol is detailed in the principal findings [19]. In brief, patients presenting to the RACPC between July 2014 and December 2017 (and then followed up through to January 2020) were screened for enrolment through assessment of cardiovascular risk factors and calculation of 5-year risk scores using the Australian Absolute Risk Calculator (cvdcheck.org.au) [19, 26] developed by the National Vascular Disease Prevention Alliance for the purpose of estimating cardiovascular event risk in primary prevention settings [27]. The calculator is based on the Framingham Risk Equation [28, 29].

Patients aged over 18 presenting to the RACPC between July 2014 and December 2017 with estimated 5-year absolute cardiovascular risk ≥ 8% were invited to participate. Additionally, ACR was categorised as low risk (8 – < 10%), intermediate risk (10 – 14%) and high risk ≥ 15%.

Exclusion criteria were known cardiac disease, pregnancy, and patients known to be at very high risk (diabetes and age > 60 years, moderate or severe chronic kidney disease, familial hypercholesterolemia, total cholesterol > 7.5 mmol/L, systolic blood pressure ≥180 mmHg or diastolic blood pressure ≥110 mmHg), where universal intensive risk management is indicated.

#### Randomisation

Patients were randomized 1:1 to best practice chest pain clinic assessment (usual care), or with the addition of an absolute risk-guided cardiovascular risk factor management strategy (intervention).

Patients allocated to the usual care group were assessed by a physician regarding their chest pain. Absolute risk scores were not discussed. Individual risk factors were discussed at the discretion of the treating clinician, consistent with standard practice in a general cardiology outpatient clinic (for example smoking cessation recommended or general practitioner follow up suggested if blood pressure or lipids elevated).

A proactive absolute risk-guided management strategy was adopted for the intervention group. In addition to chest pain assessment, patients allocated to intervention were specifically counselled regarding their 5-year cardiovascular risk score. Individual risk factors were discussed in that context, and a strategy to improve the risk score was developed. Recommendations were consistent with current primary prevention guidelines [26]. Where pharmacotherapy was indicated, this was prescribed in the clinic. Smokers were offered referral to a public smoking cessation service. All patients were provided with lifestyle advice by a registered nurse with cardiac rehabilitation experience. This review was a single-attendance clinic and participants were strongly encouraged to continue risk management strategies with their general practitioner.

### Outcomes

#### SF-6D health state utilities to assess health-related quality of life

Individual participant HSUs were generated using the SF-6D that is a prevalent multi-attribute utility instrument that uses patient-reported responses to either the SF-36 or SF-12 to derive HSUs [25]. For our study, the SF-36 questionnaire was administered in the clinic environment at baseline and at minimum 12-month follow-up.

The SF-6D health state classification system can define 18,000 potential health states, and is preferentially sensitive to both physical and psychosocial health needs when compared to other multi-attribute utility instruments that have a preponderance to either physical health (e.g. EQ-5D-5L) or psychosocial health (e.g. AQoL-8D) [30–32]. The SF-6D has been used in studies that aimed to understand the HRQoL impairments along with increasing cardiovascular disease severity for people with pre-existing cardiovascular disease [33, 34]. In this study, 11 items from the self-completed Version 1 SF-36 questionnaire were used to categorise patients in a further six domains that drive the overall HSU (namely physical functioning, role limitations, social functioning, pain, mental health and vitality), and scored using the Australian-specific utility weights obtained from a representative sample of the general population, including the Australian general population [35]. HSUs range from 0 (representing death) to 1 (representing full health) scale, with negative HSUs indicating health states that are considered to be worse than dead. The minimal important difference (MID) for the SF-6D extracted from the published literature was 0.04 utility points, where the MID represents the smallest change in HSU which is deemed to be clinically meaningful for the SF-6D [36]. The Australian general population norm for the SF-6D utility was mean 0.77 utility points. For older age cohorts namely 61–70 years and > 71 years the SF-6D population norms were 0.74 and 0.70 utility points respectively [37].

#### Sociodemographic and clinical characteristics

Sociodemographic and clinical data were also collected in the clinic environment including ACR, body mass index (BMI) estimated from height (metres (m)) and weight (kilograms (kg)) using the algorithm BMI = kg/m^2^, waist circumference (cm), serum lipids (namely LDL, HDL and total cholesterol), systolic blood pressure, smoking (yes/no) and use of antihypertensive or lipid lowering medications (yes/no).

### Statistical analyses

Descriptive statistics of mean and standard deviation (SD), median and interquartile range (IQR) were investigated for continuous variables. Counts and proportions expressed as percentages were assessed for categorical variables. Distribution of scores for SF-6D HSUs are generally not normally distributed and were checked for normality using the Shapirio-Wilk test of normality. Paired t-test, Wilcoxon-Rank or Chi-squared tests were performed, as appropriate, at the 5% level to test for significance. Linear regression analyses were also performed, to determine associations between clinical or sociodemographic variables and HSU.

Differences in between-group summary HSUs were assessed with the SF-6D’s MID of 0.04 utility points [36]. Subgroup analysis were conducted by adopting the ‘complete case’ sample approach (similar to the subgroup method adopted Sayah and colleagues that investigated the EQ-5D-5L and SF-6D for a study population of people living with Type 2 Diabetes) where individual and summary HSUs generated for the same people at baseline and follow up were examined [38].

All statistical analyses were performed using R 3.6.3 (R Foundation for Statistical Computing).

ANZCTR registration number 12617000615381 (registered 28/4/17).

## Results

### Participant characteristics

*N* = 189 participants were randomised (*n* = 98 intervention; *n* = 91 usual care) and their patient-reported responses to the SF-36 enabled the derivation of a SF-6D HSU for 96% (181/189: intervention *n* = 93, usual care *n* = 88) at baseline and 61% (115/189: intervention *n* = 63, usual care *n* = 52) at the minimum 12-month follow up mean ± SD months (control 37.8 ± 13.0 months; intervention 37.2 ± 11.8 months). The flow of patients into the study is outlined further in Supplementary Appendix A.

Table 1 provides baseline statistical comparisons of all means ± SD for clinical (including the ACR) and sociodemographic variables, and HSUs for the intervention and control groups. There were no statistical differences at baseline between the entire cohort’s intervention and usual care groups for age and sex with mean age 60 years and over two thirds of the cohorts were male. Additionally, the mean ± SD ACR score was categorised as intermediate for both groups (intervention 13.13 ± 4.34; usual care 12.77 ± 4.33 *p* = 0.56), and there were no statistical differences between the mean HSUs for the entire cohort (intervention 0.40 ± 0.26; usual care 0.45 ± 0.24, *p* = 0.12). Table 2 shows subgroup analyses for people for whom a HSU could be generated at baseline and follow-up for HSUs, and the ACR revealing no statistical differences.

**Table 1:** Comparison of sociodemographic, clinical and health state utilities for intervention and usual care at baseline

*N* = 189	Intervention *n* = 98	Usual care *n* = 91	*P* value
**SF-6D health state utility**			
*HSU*	0.40 (0.26)	0.45 (0.24)	*p* = 0.12
Mean (SD)	(*n* = 93)	(*n* = 88)	
**Socio-demographic**			
*Age* years			
* Mean (SD)*	60 (7.9)	59 (8.1)	*p = 0.65*
*Sex*			
Male	66 (67%)	71 (78%)	*p* = 0.14
Female	32 (33%)	20 (22%)	
*n* = x, %			
**Clinical risk factors**			
*ACR*			
Mean (SD)	13.13 (4.35)	12.77 (4.33)	*p* = 0.56
**Clinical**			
*BMI* kg/m^2^			
Mean (SD)	31.7 (5.6)	29.6 (5.6)	***p* = 0.008**
*Waist (cm)*			
Mean (SD)	107.3 (12.5)	103.3 (12.2)	***p* = 0.03**
*LDL (mmols/l)*			
Mean (SD)	3.43 (1.04)	3.37 (1.0)	*p* = 0.72
*Systolic BP(mmHg)*			
Mean (SD)	143 (15)	140 (13)	*p* = 0.19
*Current smoker*	49 (50%)	48 (53%)	*p* = 0.82
*Diabetes*	17 (17%)	12 (13%)	*P* = 0.55

Wilcoxon rank sum test for health state utilities

BMI and waist circumference not significant on adjustment for baseline; and for baseline and age

*ACR *Absolute risk calculator, *BMI *Body mass index, waist circumference, *LDL *Llipids, *BP* Blood pressure

**Table 2:** Statistical comparison of health state utilities (HSU) and absolute risk calculator (ACR) scores for patients for whom a utility value could be generated for baseline and follow up, including subgroup analysis where HSUs generated for the same people at baseline and follow up were examined

***N* = 189**	**Intervention** **entire cohort** **baseline** *n* = 93	**Intervention** **subgroup** **baseline** *n* = 63	**Test of significance**	**Usual care** **entire cohort** **baseline** *n* = 91	**Usual care** **subgroup** **baseline** *n* = 52	**Test of significance**
*HSU*	0.40 (0.26)	0.43 (0.27)	*p* = 0.40	0.45 (0.24)	0.47 (0.28)	*p* = 0.58
Mean (SD)	(*n* = 98)	(*n* = 62)		(*n* = 88)	(*n* = 52)	
*ACR*						
Mean (SD)	13.13 (4.35)	13.16 (4.60)	*p* = 0.97	12.77 (4.33)	12.81 (4.50)	*p* = 0.96

### Health-related quality of life using HSUs

Table 3 describes the summary statistics for the SF-6D HSUs for the intervention and usual care groups at baseline and follow-up for the entire and subgroup sample cohorts. For the entire cohort, both the intervention and usual care groups achieved a significant increase in HSUs with the intervention group reporting a higher increase for both mean (intervention 0.12 utility points, usual care 0.10 utility points) and median utilities (intervention 0.16 utility points, usual care 0.10 utility points). The difference in median HSU exceeded the MID. This was not reflected for the mean HSU.

**Table 3:** Intervention and usual care SF-6D health state utility values for the entire cohort and subgroup at baseline and follow up; and exploratory health state utilities for the stratified absolute cardiac risk calculator (ACR) for high, intermediate and low ACR at baseline and follow up (entire cohort)

***N* = 189**	**Intervention** **baseline** *n* = 98	**Intervention** **follow up** *n* = 98	Difference (baseline—follow up)	**Usual care** **baseline** *n* = 91	**Usual care** **follow-up** *n* = 91	Difference (baseline – follow up)
**Entire cohort**						
* Health state utility*						
Mean (SD)	0.40 (0.26)	0.52 (0.26)	+ 0.12	0.45 (0.24)	0.55 (0.28)	+ 0.10
Median (IQR)	0.40 (0.20 – 0.62)	0.56 (0.31–0.78)	+ 0.16^*^	0.48 (0.30 – 0.63)	0.58 (0.43–0.69)	+ 0.10^*^
Range	−0.14, 0.88	−0.09, 0.97		(−0.36; 0.95)	−0.21, 1.00	
* n* = x	(*n* = 93)	(*n* = 63)		(*n* = 88)	(*n* = 52)	
**Stratified by ACR at baseline(Entire cohort)**						
* ACR high*						
Mean (SD)	0.45 (0.26)	0.44 (0.28)	−0.01	0.46 (0.25)	0.59 (0.33)	+ 0.13
Median (IQR)	0.48 (0.35 – 0.64)	0.33 (0.22 – 0.69)	−0.15	0.53 (0.19– 0.65)	0.63 (0.48 – 0.88)	+ 0.10
Range	−0.08, 0.78	0.09 0.87		0.02, 0.79	−0.21 – 1.00	
* n* = x	(*n* = 27)	(*n* = 11)		(*n* = 17)	(*n* = 16)	
* ACR intermediate*						
Mean (SD)	0.41 (0.27)	0.59 (0.26)	+ 0.18	0.45 (0.26)	0.55 (0.27)	+ 0.10
Median (IQR)	0.46 (0.19 – 0.64)	0.62 (0.34 – 0.83)	+ 0.16^*^	0.45 (0.30– 0.63)	0.59 (0.50 – 0.69)	+ 0.14
Range	−0.14, 0.88	0.09 – 0.97		−0.36, 0.95	−0.18 – 0.93	
* n* = x	(*n* = 45)	(*n* = 23)		(*n* = 52)	(*n* = 24)	
* ACR Low*						
Mean (SD)	0.30 (0.20)	0.49 (0.24)	+ 0.19	0.45 (0.20)	0.47 (0.26)	+ 0.02
Median (IQR)	0.31 (0.14 – 0.42)	0.57 (0.34–0.64)	+ 0.26^*^	0.41 (0.36– 0.59)	0.50 (0.40 – 0.59)	+ 0.09
Range	0.01 – 0.73	−0.09—0.84		0.06, 0.84	−0.05 – 0.84	
* n* = x	(*n* = 21)	(*n* = 29)		(*n* = 19)	(*n* = 12)	
**Subgroup analyses**						
* Health state utility*						
Mean (SD)	0.43 (0.27)	0.52 (0.26)	+ 0.09	0.47 (0.28)	0.55 (0.28)	+ 0.08
Median (IQR)	0.46 (0.20 – 0.65)	0.56 (0.31–0.78)	+ 0.10	0.53 (0.29 – 0.66)	0.58 (0.43–0.69)	+ 0.05
Range	−0.14, 0.88	−0.09, 0.97		(−0.36; 0.95)	−0.21, 1.00	
* n* = x	(*n* = 63)	(*n* = 63)		(*n* = 52)	(*n* = 52)	

Wilcoxon rank test for significance at *p* < 0.05 level of significance. High ACR ≥ 15; Intermediate ACR 10–14; Low ACR < 10

^*^Indicates *p* < 0.05

Subgroup analyses reflected similar between group trends of improved HRQoL revealing a median difference between the two groups of 0.05 utility points exceeding the MID for the SF-6D (intervention 0.10 utility points; usual care 0.05 utility points). For the subgroup sample, neither intervention nor usual care groups achieved significance for mean HSU improvements (intervention 0.09 utility points *p* = 0.06; usual care 0.08 utility points *p* = 0.15).

Table 4 reports the SF-6D’s summary dimensional scores for the entire cohort. These results show there was a broad improvement in all mean dimensions with a statistically significant improvement in mean mental health and social role for the intervention group, and mental health for the usual care group. The median scores for the subdimensions revealed one point improvements for role limitations, mental health and physical function for the intervention group, and a one point improvement in the median score for mental health only was observed.

**Table 4:** SF-6D dimensional scores including physical functioning, pain, vitality; social functioning, role and mental health for the entire cohort at baseline and follow up

*N* = 189	**Intervention** **baseline** *n* = 98	**Intervention** **follow up** *n* = 98	Between-group difference	**Usual care** **Baseline** *n* = 91	**Usual care** **follow-up** *n* = 91	Between-group difference
**Dimensional scores** **Physical**						
Physical functioning						
Mean (SD)	2.99 (1.34)	2.68 (1.31)	−0.31	2.84 (1.45)	2.76 (1.56)	−0.08
Median (IQR)	3.00 (2.00 – 3.25)	2.00 (2.00 – 3.00)	−1.0	2.00 (2.00 – 3.75)	2.00 (1.25 – 4.00)	0.00
Range	1.00 – 6.00	1.00 – 6.00		(1.00- 6.00)	(1.00- 6.00)	
* n* = x	(*n* = 95)	(*n* = 68)		(*n* = 89)	(*n* = 57)	
Pain						
Mean (SD)	3.44 (1.08)	3.10 (1.33)	−0.33	3.56 (1.21)	3.24 (1.37)	−0.32
Median (IQR)	3.00 (3.00 – 4.00)	3.00 (2.00 – 3.00)	0.0	3.00 (3.00 – 4.00)	3.00 (2.00 – 4.00)	0.00
Range	1.00 – 6.00	1.00 – 6.00		(1.00- 6.00)	(1.00- 6.00)	
* n* = x	(*n* = 96)	(*n* = 69)		(*n* = 90)	(*n* = 57)	
Vitality						
Mean (SD)	3.34 (1.07)	3.17 (0.94)	−0.17	3.11 (1.05)	2.82 (0.98)	−0.29
Median (IQR)	3.00 (3.00 – 4.00)	3.00 (3.00 – 4.00)	0.0	3.00 (2.00 – 4.00)	3.00 (2.00 – 3.00)	0.00
Range	1.00 – 5.00	1.00 – 5.00		1.00 – 5.00	(1.00- 5.00)	
* n* = x	(*n* = 96)	(*n* = 68)		(*n* = 90)	(*n* = 57)	
**Dimensional scores ** **Physical**						
Social functioning						
Mean (SD)	2.45 (1.16)	2.30 (1.21)	−0.15	2.19 (1.06)	2.07 (1.06)	−0.12
Median (IQR)	2.00 (1.00 – 3.00)	2.00 (1.00 – 3.00)	0.0	2.00 (1.00 – 3.00)	2.00 (1.00 – 3.00)	0.00
Range	1.00 – 5.00	1.00 – 5.00		1.00 – 5.00	1.00 – 5.00	
* n* = x	(*n* = 95)	(*n* = 69)		(*n* = 89)	(*n* = 57)	
Role						
Mean (SD)	2.40 (1.31)	1.93 (1.12)	−0.47	2.36 (1.21)	2.22 (1.27)	−0.14
Median (IQR)	2.00 (1.00 – 4.00)	1.00 (1.00 – 3.00)	−1.0^*^	2.0 (1.0 – 3.0)	2.0 (1.0 – 3.5)	0.00
Range	1.00 – 4.00	1.00 – 4.00		1.0 – 4.0	1.0 – 4.0	
* n* = x	(*n* = 94)	(*n* = 66)		(*n* = 89)	(*n* = 57)	
Mental health						
Mean (SD)	2.87 (1.15)	2.45 (1.17)	−0.42	2.62 (1.04)	2.25 (1.21)	−0.40
Median (IQR)	3.00 (2.00 – 4.00)	2.00 (1.50 – 3.00)	−1.0^*^	3.0 (2.0 – 3.0)	2.0 (1.0 – 3.0)	−1.0^*^
Range	1.00, 5.00	1.00, 5.00		1.0, 5.0	1.0, 5,0	
* n* = x	(*n* = 96)	(*n* = 66)		(*n* = 90)	(*n* = 57)	

^*^Indicates *p* < 0.05

### Health state utility values by stratified high, intermediate and low ACR

Table 3 also reports the *exploratory* examination of summary HSUs for the patients’ ACR stratified into high, intermediate and low risk categories (according to the inclusion criteria of 5-year absolute risk > 8%) for the entire cohort sample only. The results are exploratory due to the relatively small sample sizes of the stratified scores (particularly for the follow up summary HSUs). The intervention group generated a statistically significant increase in mean HSUs for patients in the intermediate and low risk ACR categories, and HSUs remained stable for patients who were in the high-risk category (ACR ≥ 15%). Divergent results were revealed for the usual care group where patients in the high-risk category reported an increased HSU at follow up, however, this result was not statistically significant (Table 3).

## Discussion

To our knowledge, this is the first detailed study to investigate the quantitative HRQoL impacts of proactive absolute cardiovascular risk management in a hospital-based clinic. We found that HRQoL increased more for people who received absolute cardiovascular risk intervention compared to usual care (Table 3). The between-group median difference exceeded the minimal important difference for the intervention group indicating a clinically meaningful difference between the two management strategies. This suggests that patient-centred tailored proactive absolute cardiac risk counselling within the RACPC environment may yield clinically meaningful change. The difference was also robust to subgroup analysis.

### Overall HSUs and health-related quality of life

Examination of the health preferences literature using the SF-6D for an Australian CVD cohort revealed one study of a *chronic heart disease* cohort that investigated a head-to-head comparison of HSUs for the EQ-5D-3L and SF-6D multi-attribute utility instruments for ‘Young@Heart Study’ patients with a mean age of 70 years and hospitalised with chronic heart disease [34]. The study recommended the use of the SF-6D (compared to the EQ-5D-3L) in mild CVD conditions. In contrast to our study that investigated a cardio-preventative cohort (no known cardiac disease yet increased cardiac risk factors), the Young@Heart study did not use the Australian value set to estimate the HSUs—instead using the UK value sets for both instruments to enable comparisons. Moreover, the Australian Young@Heart study was for a cohort with established cardiovascular disease [34]. Our study is the first to investigate an Australian cardio-preventative cohort using SF-6D HSUs.

Our study established that HRQoL improved more for patients who received proactive absolute cardiac risk counselling and this difference was clinically meaningful for the median values. We noted that the difference was not clinically meaningful for the mean changes, nevertheless, the trend revealed a between group improvement for the intervention. Potential factors driving this change may be patient engagement in lifestyle changes that were systematically discussed in a patient-centred ACR counselling session for the intervention group with the aim of improving smoking cessation, blood pressure and lipid profiles, increased physical activity and weight loss. SF-6D dimensional scores also suggested increased patient engagement through significant improvements in mental health and social role for the intervention group. Proactive ACR counselling about modifiable lifestyle factors including increased physical activity, smoking cessation and weight loss could be driving these dimensional improvements. Overall the findings are exploratory and our conclusions call for a larger confirmatory study.

### Divergence of HSUs with stratified ACR for intervention and usual care

An additional exploratory finding was the investigation of summary HSU changes for stratified absolute cardiac risk scores. Patients in the intervention group’s high cardiac risk category recorded a stable HRQoL whereas those in the usual care group’s high cardiac risk category recorded a counter-intuitive increased HRQoL.

A recent systematic review that examined the impact of the provision of cardiovascular disease risk estimates to healthcare professionals and patients concluded that the challenges to the communication of risk are well known and that further research is required to better understand these challenges [9]. Another study examined the use of effective communication by clinicians to convey cardiac risk information to patients and concluded that effective communication strategies translate to improved accuracy of cardiac risk perception and subsequent improved uptake of cardioprotective measures [39]. A further study that examined the influence of risk perception, risk preferences and information processing on cardiovascular risk counselling found that high-risk individuals ranked by biomarkers (e.g., obese, diabetes or hypertension) set a target risk lower than others by about 1% point, potentially reflecting an over-optimism bias in this group [10]. Importantly, this study concluded that given the global pandemic of CVD, there are public health gains to be made from personalised risk communication if it is better tailored to account for individuals’ preferences and risk perception [10].

Our exploratory findings have attempted to answer the call to provide an interventional strategy to implement formal and tailored CVD calculation into consultation [9]. This study embedded the SF-6D multi-attribute utility instrument into a clinical trial to improve understanding of the impacts on HRQoL regarding the communication of absolute cardiac risk. These results suggest that when patients are provided with proactive, consistent, tailored and effectively communicated absolute cardiac risk there could be an enhanced understanding of this risk, leading to the adoption of strategies to improve risk profile. The intervention group revealed a significant increase in HSUs for low and intermediate risk categories – suggesting that patients responded positively to ACR counselling and management. Moreover, the HSUs for people in the high-risk ACR category remained stable. On the other hand, the usual care group’s HSUs in the high cardiac risk category increased at follow up (albeit not statistically significant). This may reflect a lack of insight into cardiovascular risk within the control group, however, we caution that the finding is exploratory and requires further evaluation in a larger confirmatory study.

Both doctors and patients have been found to inaccurately estimate cardiovascular risk in a primary care setting [40], with a tendency towards systematic under-estimation of risk – so-called ‘optimistic bias’ [41]. It has been recommended that future studies develop strategies to implement formal CVD risk calculation into consultation and test the strategies in actual consultations [40]. There may be additional benefit in adding a generic and preferentially sensitive HRQoL assessment tool to ACR assessment to investigate HSUs and the drivers of these HSUs particularly through the psychosocial dimensions of health.

### COVID-19: the role of RACPC’s

Since we conducted our clinical RACPC evaluations [17, 19], and now this detailed HRQoL study regarding the benefits of proactive cardiac risk counselling for a cardio-preventative cohort that has presented to a RACPC, COVID-19 has resulted in millions of deaths worldwide particularly for people with cardiac risk factors [42]. The emergence of Long COVID is also set to take an additional toll on an already burdened healthcare system [43, 44].

During the ongoing COVID-19 pandemic, there may be a particular role for RACPC clinics through reducing emergency department re-attendances and facilitating opportunistic management of cardiovascular risk factors. Optimizing cardiovascular health may reduce health system utilization and may also prevent some of the serious morbidity associated with COVID-19 infection.

### Integrating patient reported outcomes in clinical practice

The International Society for Quality of Life Research has developed a clinical users guide to encourage the routine collection of patient reported outcomes which “are rarely collected in routine clinical practice” [24]. Recent evidence has also found that integrating patient-reported outcomes in clinical practice has the potential to enhance patient-centred care, including for people with complex risk factors that can be modified with lifestyle changes [45, 46]. Within this broader and evolving context of patient-centredness in clinical care, our study has highlighted the clinical relevance of multi-attribute utility instrument and HSU analyses in the cardioprotective clinical setting. There may be a role within RACPCs for the adoption of a generic and preferentially sensitive multi-attribute utility instrument in routine clinical care. Evaluation of the clinical predictive utility of the instrument in this setting is required.

### Strengths and limitations

A randomised control trial study design was a strength for our health preferences study. Use of the SF-6D multi-attribute utility instrument was a strength given the instrument’s sensitivity in other CVD populations. Nevertheless, use of a multi-attribute utility instrument that is preferentially sensitive to psychosocial health (such as the AQoL-8D or the recently released SF-6Dv2 [47]) may have revealed additional information regarding the individual dimensions of psychosocial health (and some cues regarding enhanced patient understanding). The main weakness of our study was the incomplete SF-36 data for almost one-third of the cohort at the follow up timepoint. However, we also conducted subgroup analysis and this methodology is also reflected in other studies [38].

## Conclusions

Challenges regarding the communication of ACR are well-known. Our HRQoL study has established that proactive ACR guided management in the RACPC improves HRQoL.

We recommend a larger confirmatory study with increased follow-up to particularly investigate HSUs with stratified ACR using both the SF-6D and the AQoL-8D instrument (which has a more detailed evaluation of psychosocial health) to further assess the impact of patient behaviour through HSUs and super and individual dimensional scores and the predictive capabilities of the patient-reported outcome measures.

## Supplementary Information

Supplementary Material 1.

## Data Availability

No datasets were generated or analysed during the current study.
