# Supplementary material for: Evaluation of health-related quality of life changes in an Australian rapid access chest pain clinic
Source: BMC Health Serv Res. 2025 Jan 2;25:8. doi: 10.1186/s12913-024-12135-0 (PMC11697740; doi:10.1186/s12913-024-12135-0)

**Supplementary Figure 1:** Individual health state utilities (HSU) for people who attended a Rapid Access Chest Pain Clinic with new onset chest pain with no cardiac cause but increased cardiac risk for the intervention and control groups for the entire sample at baseline and follow up.


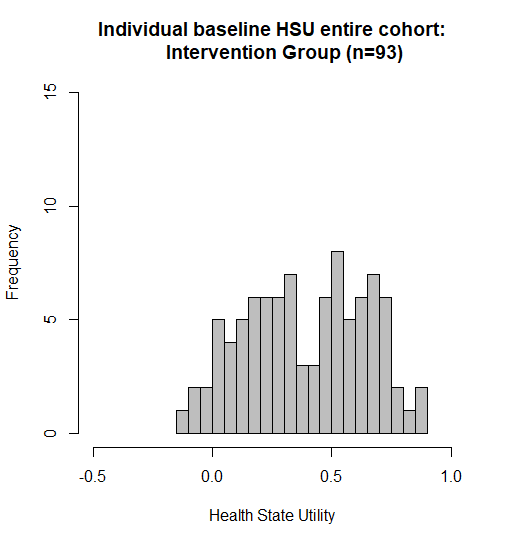

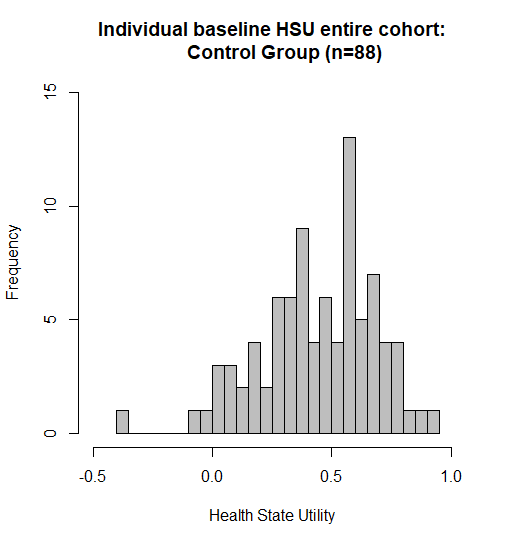

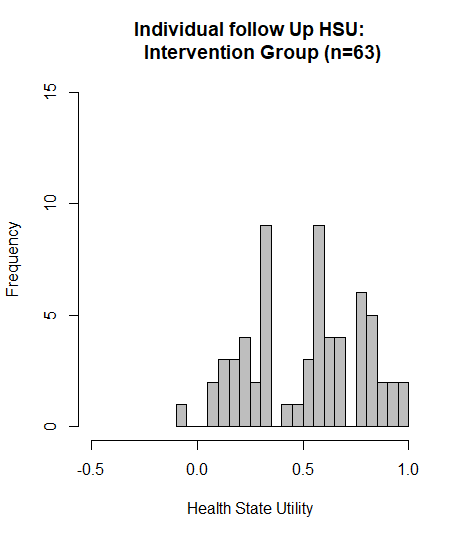

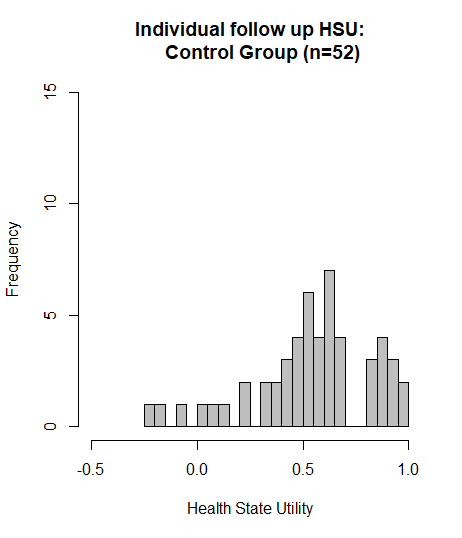

Supplement: Supplementary file 1 — Supplementary Material 1. [file 12913_2024_12135_MOESM1_ESM.docx]
